# Correction to “Discrepant results of the total pacing prematurity in orthodromic reciprocating tachycardia with right bundle branch block”

**DOI:** 10.1002/joa3.12979

**Published:** 2023-12-29

Mizutani A, Okada M, Miyazaki N, Tanaka K, Tanaka N. Discrepant results of the total pacing prematurity in orthodromic reciprocating tachycardia with right bundle branch block. J Arrhythmia. 2023;39:973–976. https://doi.org/10.1002/joa3.12933.

References have been reordered as follows and the citations are revised in the article

1. Michaud GF, Tada H, Chough S, Baker R, Wasmer K, Sticherling C, et al. Differentiation of atypical atrioventricular node re‐entrant tachycardia from orthodromic reciprocating tachycardia using a septal accessory pathway by the response to ventricular pacing. J Am Coll Cardiol. 2001;38:1163–67.
2. González‐Torrecilla E, Arenal A, Atienza F, Osca J, García‐Fernández J, Puchol A, et al. First postpacing interval after tachycardia entrainment with correction for atrioventricular node delay: a simple maneuver for differential diagnosis of atrioventricular nodal reentrant tachycardias versus orthodromic reciprocating tachycardia. Heart Rhythm. 2006;3:674–79.
3. Kaiser DW, Hsia HH, Dubin AM, Liem LB, Viswanathan MN, Zei PC, et al. The precise timing of tachycardia entrainment is determined by the postpacing interval, the tachycardia cycle length, and the pacing rate: theoretical insights and practical applications. Heart Rhythm. 2016;13:695–703.
4. Maruyama M, Uetake S, Miyauchi Y, Seino Y, Shimizu W. Analyses of the mode of termination during diagnostic ventricular pacing to differentiate the mechanisms of supraventricular tachycardias. JACC Clin Electrophysiol. 2017;3:1252–61.
5. AlMahameed ST, Buxton AE, Michaud GF. New criteria during right ventricular pacing to determine the mechanism of supraventriculartachycardia. Circ Arrhythm Electrophysiol. 2010;3:578–84.
6. Boonyapisit W, Methavigul K, Krittayaphong R, Sriratanasathavorn C, Pumprueg S, Suwanagool A, et al. Determining the site of accessory pathways in orthodromic reciprocating tachycardia by using the response to right ventricular pacing. Pacing Clin Electrophysiol. 2016;39:115–21.

We apologize for this error.

